# Comparative genomics reveals novel genes associated with sensory cilia

**DOI:** 10.1186/2046-2530-4-S1-P16

**Published:** 2015-07-13

**Authors:** B Piasecki, T Sasani, B O'Flaherety, T Swords, J Henriksson, E De Stasio, P Swoboda

**Affiliations:** 1 Biology, Lawrence University, Appleton, WI, USA; 2 Department of Biosciences and Nutrition, Karolinska Institute, Huddinge, Sweden

## Objective

To identify evolutionarily conserved ciliary genes that facilitate sensory-specific roles.

## Methods

Using reciprocal BLAST analyses, the genomes of organisms that do not make cilia (the plant *Arabidopsis thaliana *and the yeast *Saccharomyces cerevisiae*) and that retain motile but not sensory cilia (the moss *Physcomitrella patens*) were subtracted from the genomes of organisms that have retained sensory cilia (the worm *Caenorhabditis elegans *and the alga *Chlamydomonas reinhardtii*).

## Results

These analyses revealed a list of 272 genes that are found exclusively in organisms with sensory cilia but not motile cilia. Importantly, nearly 10% of the genes on this list have previously been implicated in sensory cilia-specific roles, thus providing numerous internal positive controls that demonstrate this list is enriched with sensory-specific ciliary genes. A subset of uncharacterized candidate genes are currently being studied in *C. elegans*, which retains cilia exclusively on a set of neurons termed ciliated sensory neurons (CSNs). We are currently generating a number of promoter- and gene-to-green fluorescent protein (GFP) fusion constructs in order to determine the expression and localization patterns of the proteins encoded by these genes. Two of these candidate genes, which are found in **w**orms (*C. elegans*) and **a**lgae (*C. reinhardtii*) but not in **m**oss (*P. patens*) have been termed *wam-1 *and *wam-2*, respectively. Expression of *wam-1 *appears to be localized exclusively in the support cells of ciliated dopaminergic neurons, while expression of *wam-2 *has yet to be fully characterized.

## Conclusion

Our analyses have successfully revealed novel genes involved in ciliary sensory-specific processes in animals.

